# Association between hearing aid use and cognitive function among Chinese middle-aged and older adults with hearing impairment: the mediating effect of social activity

**DOI:** 10.7189/jogh.16.04120

**Published:** 2026-05-22

**Authors:** Xinyi Wang, Xianger Gu, Sunxi Mao, Zinan Zhang, Zhonghua Wang

**Affiliations:** 1 School of Health Policy and Management, Nanjing Medical University, Nanjing, China; 2 Laboratory for Digital Intelligence and Health Governance, Nanjing Medical University, Nanjing, China

## Abstract

**Background:**

In China’s ageing population, hearing-impaired middle-aged and older adults face cognitive vulnerability. We aimed to explore the effects of hearing aid use on cognitive function and to examine the mediating role of social activity in this relationship.

**Methods:**

We sourced data from the China Health and Retirement Longitudinal Study for the years 2011, 2013, 2015, 2018, and 2020. Using multiple linear regression and binary logistic regression, we analysed the association between hearing aid use and cognitive scores, as well as the risk of cognitive impairment in middle-aged and older adults with hearing impairments. PROCESS models explored whether social activities mediated the relationship between hearing aid use and cognitive function.

**Results:**

Hearing aid use was significantly positively associated with cognitive performance among middle-aged and older adults with hearing impairment (coefficient = 1.226). Hearing aid use was also significantly associated with a 29.6% lower risk of cognitive impairment compared with nonusers within this population (odds ratio = 0.704). Moreover, hearing aid use was associated with higher cognitive scores and a reduced risk of cognitive impairment, linked to increased participation in social activities, particularly intellectual and physical pursuits. Finally, the direct associations between hearing aid use and cognitive function, and the mediating role of social activities in this relationship, varied by gender and age.

**Conclusions:**

Promoting hearing aid use and social activity is vital to improve cognitive function and reduce impairment risk in middle-aged and older adults with hearing loss. Tailored interventions should address gender- and age-related differences to maximise benefits.

Cognitive impairment – decline in memory, attention, language, executive and calculative functions – afflicts >55 million older adults globally [1]. China adds 0.36 million new cases yearly, projected to reach 48.7 million by 2060 [2], compressing healthy longevity and overburdening healthcare and social-care systems [3]. Health China 2030 accordingly designates dementia prevention as a core healthy-ageing indicator, prescribing individual/family, governmental and societal interventions [4]. Longitudinal neuroimaging now shows hippocampal atrophy accelerates after the age of 45 years, implicating mid-life as the critical window for preservation strategies [5–7]. Therefore, the cognitive health of middle-aged and older adults deserves considerable attention.

Hearing impairment robustly accelerates cognitive decline. Pooled evidence shows that affected adults have a 1.9-fold higher risk of developing cognitive impairment, and even mild loss forecasts long-term dementia among the initially cognitively intact [8,9]. Beyond peripheral deficits, concomitant cognitive decline impairs sound perception and central auditory processing, while central dysfunction, in turn, exacerbates hearing deficits, locking the two domains in a self-reinforcing cycle [10,11]. These findings underscore that individuals with hearing impairment are particularly vulnerable to cognitive decline.

A hearing aid amplifies sound to restore audibility and improve speech perception [12], thereby reducing cognitive load and sensory deprivation [13,14]. Cross-sectional studies from the USA [9] and UK [15] have also reported an association between hearing aid use and enhanced cognitive performance among older adults. In the current research landscape, the relationship between hearing aid utilisation and cognitive function has primarily been examined in older populations. Consequently, there has been a notable lack of focus on directed towards middle-aged and older adults experiencing hearing impairment. This research gap may limit the effectiveness of interventions to address cognitive decline, particularly during the critical transitional period from middle to late adulthood.

Social activity, defined as engagement in personal, community, or civic networks [16,17], presents unique challenges for individuals with hearing impairments, who consistently experience increased listening effort. Lavie’s cognitive load theory [18] suggests that when dual tasks are undertaken simultaneously in high cognitive load conditions for the primary auditory task, performance on the secondary social activity task is likely to decline if processing capacity is exceeded. Nonetheless, substantial evidence correlates increased social engagement with the preservation of instrumental activities of daily living (IADL) [19], reduced depression rates [20] and a 30–50% decrease in dementia risk [21,22]. More importantly, the reduced social activity may also cause specific damage to cognitive function. Research indicates that active involvement in social activities provides crucial, complex environmental stimuli to the brain, which are essential for sustaining cognitive reserves [23]. Therefore, when hearing loss reduces social participation, it can significantly impair cognitive function [24]. By improving auditory capabilities, hearing aids mitigate the barriers and pressures associated with social activity, enabling individuals to reintegrate into active social lives [25-29]. Furthermore, ongoing active participation in social activities may positively affect cognitive function. Therefore, we propose that social activity mediates the relationship between hearing aid use and cognitive function.

Amid China’s rapid ageing, prior evidence suggests that the cognitive vulnerability of hearing-impaired middle-aged and older adults warrants closer examination. Utilising nationally representative data, we assessed the effect of hearing aid use on the cognitive function of this demographic and explored whether social activity mediates these benefits. Considering gender differences and the fact that 65 years of age serves as a key demographic and policy marker of ageing in China, we conducted heterogeneity analyses by gender and age. This research carries important implications for developing targeted interventions and public health strategies designed to alleviate cognitive decline among the ageing population.

## METHODS

### Data source

In this study, we employed data from the China Health and Retirement Longitudinal Study (CHARLS) [30] covering the period from 2011 to 2020. We pooled data from five waves and, to maintain the independence of observations, retained only the first available survey entry for each participant, creating a repeated cross-sectional analytic sample. The analysis centred on a cohort of 3,08 respondents aged ≥45 years who reported experiencing hearing impairments, thereby excluding those with normal hearing or incomplete data. The sample sizes for each wave were as follows: 1120 participants in 2011, 612 in 2013, 808 in 2015, 819 in 2018, and 449 in 2020. We gathered the CHARLS datasets [30] using a multistage probability sampling method that included participants from approximately 10 000 households across 450 villages and 150 counties. For the analysis, we used the GRABDROP guidelines for (Table S1 in the **Online Supplementary Document**) [28].

### Cognitive function

We quantified cognitive function with the 31-point Health and Retirement Study battery – orientation (0–5), memory (0–20), computation (0–5) and drawing (0–1) – where higher scores indicated better cognition [29,31]. We defined impairment by the ageing-associated cognitive decline criteria. Within each 10-year age band of ≥45 years, participants scoring less than the mean minus one standard deviation of their age-and-education (illiterate *vs.* non-illiterate) were classified as impaired [32,33]. It is important to note that we recalculated the cut-off scores separately for each survey wave to ensure accurate prevalence estimates across cohorts.

### Hearing aid use

We assessed the hearing aid use through the question, ‘Have you ever worn a hearing aid?’ A response of ‘0’ (no) indicated non-use, while a response of ‘1’ (yes) indicated use. This binary classification enabled a clear assessment of hearing aid prevalence in the participant cohort.

### Social activity

We classified social activity into three aspects in terms of the CHARLS questionnaire: social interaction (with friends and providing assistance to family, friends, or neighbours), intellectual (participation in games of intellect, such as mahjong, chess, card games, and participation in community spaces) and physical activity (participation in activities such as dancing, exercise, and Qigong). In this study, we categorised social activity into two groups: ‘0 ‘for ‘no’ and ‘1’ for ‘yes,’ based on responses to the original questionnaire item, ‘Are you doing any of these activities in the last month?’

### Other control variables

We grouped the control variables into the categories of demographics, health, and lifestyle. Demographics comprised age, sex, education, marital status (married *vs.* other), and household per-capita consumption expenditure, expressed in four ascending bands (CNY <5000, 5000–9999, 10 000–19 999, and ≥20 000). Health status combined four indicators: self-rated health (very good, good, fair, poor, very poor), chronic diseases (none, one, multiple), instrumental IADL operationalised as a 0–5 count of difficulties in housework, cooking, shopping, money management, and medication adherence, and depression dichotomised at a Centre for Epidemiologic Studies Depression Scale 10 item cut-off of 10. Lifestyle variables included smoking and drinking.

### Statistical analysis

To investigate the relationship between hearing aid utilisation and cognitive scores, we initially conducted multiple linear regression analyses among middle-aged and older adults with hearing impairments. Subsequently, we assessed the impact of hearing aid usage on the risk of cognitive impairment within this demographic using binary logistic regression. Furthermore, we employed the three-step regression approach proposed by Baron and Kenny to examine the mediating effect of social activities on the association between hearing aid use and cognitive function. All statistical analyses were performed using Stata, version 17 (StataCorp, College Station, Texas, USA). To address the issue of multiple testing, we applied the Bonferroni correction, setting the significance threshold at *P*-value <0.1.

## RESULTS

### Descriptive statistics

We included 3808 Chinese middle-aged and older adults with hearing impairment, among whom 18.17% exhibited cognitive impairment, and 6.64% used a hearing aid. Among the respondents, 16.41% were aged 45–54 years, and 20.40% were aged ≥75 years. Furthermore, 47.74% were women, and 36.66% participated in social interactions. Moreover, 15.28% participated in intellectual activities, and 5.41% in physical activities (**Table 1**).

**Table 1:** Description of the sample, n (%)

	Year					
**Characteristics**	**2011**	**2013**	**2015**	**2018**	**2020**	**Total**
Cognitive impairment						
*No*	910 (81.25)	523 (85.46)	668 (82.67)	631 (77.05)	384 (85.52)	3116 (81.83)
*Yes*	210 (18.75)	89 (14.54)	140 (17.33)	188 (22.95)	65 (14.48)	692 (18.17)
Hearing aid use						
*No*	1059 (94.55)	569 (92.97)	767 (94.93)	742 (90.60)	418 (93.10)	3555 (93.36)
*Yes*	61 (5.45)	43 (7.03)	41 (5.07)	77 (9.40)	31 (6.90)	253 (6.64)
Age in years						
*45–54*	162 (14.46)	95 (15.52)	200 (24.75)	159 (19.41)	9 (2.00)	625 (16.41)
*55–64*	377 (33.66)	215 (35.13)	246 (30.45)	265 (32.36)	62 (13.81)	1165 (30.60)
*65–74*	327 (29.20)	212 (34.64)	251 (31.06)	266 (32.48)	185 (41.20)	1241 (32.59)
*≥75*	254 (22.68)	90 (14.71)	111 (13.74)	129 (15.75)	193 (42.98)	777 (20.40)
Gender						
*Female*	529 (47.23)	294 (48.04)	413 (51.11)	418 (51.04)	164 (36.53)	1818 (47.74)
*Male*	591 (52.77)	318 (51.96)	395 (48.89)	401 (48.96)	285 (63.47)	1990 (52.26)
Marital status						
*Other*	244 (21.79)	103 (16.83)	139 (17.20)	135 (16.48)	108 (24.05)	729 (19.14)
*Married*	876 (78.21)	509 (83.17)	669 (82.80)	684 (83.52)	341 (75.95)	3079 (80.86)
Education						
*Non-illiterate*	798 (71.25)	461 (75.33)	604 (74.75)	661 (80.71)	307 (68.37)	2831 (74.34)
*Illiterate*	323 (28.75)	151 (24.67)	204 (25.25)	158 (19.29)	142 (31.63)	977 (25.66)
Household per capita consumption expenditure in CNY						
*<5000*	661 (59.02)	173 (28.27)	224 (27.72)	153 (18.68)	95 (21.16)	1306 (34.30)
*5000–9999*	289 (25.80)	227 (37.09)	227 (28.09)	241 (29.43)	121 (26.95)	1105 (29.02)
*10 000–19 999*	129 (11.52)	141 (23.04)	235 (29.08)	253 (30.89)	129 (28.73)	887 (23.29)
*≥20 000*	41 (3.66)	71 (11.60)	122 (15.10)	172 (21.00)	104 (23.16)	510 (13.39)
Self-rated health						
*Very good*	43 (3.84)	23 (3.76)	63 (7.80)	53 (6.47)	39 (8.69)	221 (5.80)
*Good*	127 (11.34)	55 (8.99)	69 (8.54)	50 (6.11)	52 (11.58)	353 (9.27)
*Fair*	477 (42.59)	296 (48.37)	380 (47.03)	387 (47.25)	219 (48.78)	1759 (46.19)
*Poor*	366 (32.68)	186 (30.39)	213 (26.36)	246 (30.04)	99 (22.05)	1110 (29.15)
*Very poor*	107 (9.55)	52 (8.50)	83 (10.27)	83 (10.13)	40 (8.91)	365 (9.59)
Chronic disease						
*None*	246 (21.96)	120 (19.61)	185 (22.90)	91 (11.11)	62 (13.81)	704 (18.49)
*One*	315 (28.13)	134 (21.90)	175 (21.66)	143 (17.46)	102 (22.72)	869 (22.82)
*Multiple*	559 (49.91)	358(58.50)	448 (55.45)	585 (71.43)	285 (63.47)	2235 (58.69)
IADL						
*0 functional impairment*	648 (57.86)	387 (63.24)	527 (65.22)	525 (64.10)	323 (71.94)	2410 (63.29)
*1 functional impairment*	167 (14.91)	101 (16.50)	109 (13.49)	126 (15.38)	47 (10.47)	550 (14.44)
*2 functional impairment*	113 (10.09)	59 (9.64)	64 (7.92)	59 (7.20)	33 (7.35)	328 (8.61)
*3 functional impairment*	86 (7.68)	29 (4.74)	44 (5.45)	61 (7.45)	19 (4.23)	239 (6.28)
*4 functional impairment*	67 (5.98)	23 (3.76)	37 (4.58)	29 (3.54)	18 (4.01)	174 (4.57)
*5 functional impairment*	39 (3.48)	13 (2.12)	27 (3.34)	19 (2.32)	9 (2.00)	107 (2.81)
Depression						
*No*	526 (46.96)	347 (56.70)	413 (51.11)	404 (49.33)	251 (55.90)	1941 (50.97)
*Yes*	594 (53.04)	265 (43.30)	395 (48.89)	415 (50.67)	198 (44.10)	1867 (49.03)
Smoke						
*No*	597 (53.30)	332 (54.25)	439 (54.33)	449 (54.82)	184 (40.98)	2001 (52.55)
*Yes*	523 (46.70)	280 (45.75)	369 (45.67)	370 (45.18)	265 (59.02)	1807 (47.45)
Drink						
*No*	752 (67.14)	381 (62.25)	540 (66.83)	534 (65.20)	296 (65.92)	2503 (65.73)
*Yes*	368 (32.86)	231 (37.75)	268 (33.17)	285 (34.80)	153 (34.08)	1305 (34.27)
Social interaction						
*No*	765 (68.30)	372 (60.78)	483 (59.78)	487 (59.46)	305 (67.93)	2412 (63.34)
*Yes*	355 (31.67)	240 (39.22)	325 (40.22)	332 (40.54)	144 (32.07)	1396 (36.66)
Intellectual activity						
*No*	963 (85.98)	505 (82.52)	665 (82.30)	694 (84.74)	387 (86.19)	3226 (84.72)
*Yes*	157 (14.02)	107 (17.48)	143 (17.70)	125 (15.26)	62 (13.82)	582 (15.28)
Physical activity						
*No*	1082 (96.61)	567 (92.65)	754 (93.32)	772 (94.26)	427 (95.10)	3602 (94.59)
*Yes*	38 (3.39)	45 (7.35)	54 (6.68)	47 (5.74)	22 (4.90)	206 (5.41)

IADL – instrumental activities of daily living

### Multivariate regression results

The results indicate a significant positive association between hearing aid use and cognitive scores (coefficient = 1.23), whereas each successive age decade was significantly associated with lower scores (coefficient = –1.66 for age 55–64 years; coefficient = –2.81 for age 65–74 years; and coefficient = –4.74 for age ≥75 years). Men significantly outperformed women (coefficient = 2.20), and illiterate respondents scored significantly lower (coefficient = –2.20). Higher IADL, being married, greater household expenditure, absence of chronic disease and non-depression were also significantly associated with better cognitive scores (**Table 2**).

**Table 2:** Multiple linear regression of cognitive scores among middle-aged and older adults with hearing impairment

Variables	Coefficient (95% CI)	*P*-value
Hearing aid use		
*No*	ref	
*Yes*	1.226 (0.526, 1.927)	0.001
Age in years		
*45–54*	ref	
*55–64*	–1.660 (–2.199, –1.120)	<0.001
*65-74*	–2.812 (–3.275, –2.349)	<0.001
*≥75*	–4.744 (–5.385, –4.103)	<0.001
Gender		
*Female*	ref	
*Male*	2.200 (2.194, 3.205)	<0.001
Marital status		
*Other*	ref	
*Marry*	1.567 (1.079, 2.054)	<0.001
Education		
*Non-illiterate*	ref	
*Illiterate*	–2.203 (–2.630, –1.770)	<0.001
Household per capita consumption expenditure in CNY		
*<5000*	ref	
*5000–9999*	0.897 (0.447, 1.347)	<0.001
*10000-19999*	1.387 (0.892, 1.881)	<0.001
*≥20000*	2.373 (1.777, 2.970)	<0.001
Self-rated health		
*Very good*	ref	
*Good*	–0.045 (–0.965, 0.874)	0.923
*Fair*	0.365 (–0.406, 1.137)	0.354
*Poor*	–0.230 (–1.057, 0.596)	0.585
*Very poor*	–0.080 (–1.052, 0.892)	0.871
Chronic disease		
*None*	ref	
*One*	0.491 (-0.055, 1.038)	0.078
*Multiple*	1.276 (0.784, 1.769)	<0.001
IADL		
*0 functional impairment*	ref	
*1 functional impairment*	–0.776 (–1.291, –0.261)	0.003
*2 functional impairment*	–1.848 (–2.502, –1.194)	<0.001
*3 functional impairment*	–2.731 (–3.490, –1.972)	<0.001
*4 functional impairment*	–3.396 (–4.270, –2.523)	<0.001
*5 functional impairment*	–5.114 (–6.213, –4.015)	<0.001
Depression		
*No*	ref	
*Yes*	–1.280 (–1.661, –0.899)	<0.001
Smoke		
*No*	ref	
*Yes*	–0.339 (–0.817, 0.138)	0.163
Drink		
*No*	ref	
*Yes*	0.128 (–0.279, 0.536)	0.538
Year		
*2011*	ref	
*2013*	0.234 (–0.318, 0.786)	0.406
*2015*	–0.143 (–0.657, 0.372)	0.587
*2018*	–0.934 (–1.462, –0.406)	<0.001
*2020*	0.359 (–0.281, 0.999)	0.271
Constant	11.507 (10.459, 12.555)	<0.001
		0.001

CI – confidence interval, IADL – instrumental activities of daily living, ref – reference

A suggestive association was found between hearing aid use and a lower likelihood of cognitive impairment (odds ratio (OR) = 0.704), suggesting that middle-aged and older adults with hearing impairment who used hearing aids had a 29.6% lower relative risk of cognitive impairment compared to those who did not. The risk of cognitive impairment was higher among individuals aged 55–64 and 65–74 years, increasing by 28.90% and 32.60%, respectively, compared with those aged 45–54 (OR = 1.289, 1.326). Additionally, men exhibited a 62.60% lower risk than women (OR = 0.374), and married individuals with hearing impairments had a 33.1% lower risk compared to those with other marital statuses (OR = 0.669). Individuals with higher household per capita consumption expenditure (CNY 5000–9999, CNY 10 000–19 999, and CNY>20 000) were associated with a 25.40%, 19.50%, and 47.40% lower risk of cognitive impairment, respectively. Conversely, those with IADL disorders were associated with an increased risk of cognitive impairment compared to those without such disorders (**Table 3**).

**Table 3:** Binary-logistic regression of cognitive impairment among middle-aged and older adults with hearing impairment

Variables	OR (95% CI)	*P*-value
Hearing aid use		
*No*	ref	
*Yes*	0.704 (0.464, 1.068)	0.099
Age in years		
*45–54*	ref	
*55–64*	1.289 (0.973, 1.708)	0.077
*65–74*	1.326 (0.996, 1.766)	0.054
*≥75*	0.800 (0.568, 1.125)	0.199
Gender		
*Female*	ref	
*Male*	0.374 (0.287, 0.487)	<0.001
Marital status		
*Other*	ref	
*Married*	0.669 (0.529, 0.847)	0.001
Education		
*Non-illiterate*	ref	
*Illiterate*	1.478 (1.180, 1.852)	0.001
Household per capita consumption expenditure in CNY		
*<5000*	ref	
*5000–9999*	0.746 (0.596, 0.934)	0.011
*10 000–19 999*	0.805 (0.628, 1.032)	0.087
*≥20 000*	0.526 (0.379, 0.730)	<0.001
Self-rated health		
*Very good*	ref	
*Good*	1.524 (0.920, 2.534)	0.102
*Fair*	1.120 (0.721, 1.739)	0.614
*Poor*	1.436 (0.907, 2.272)	0.122
*Very poor*	1.139 (0.678, 1.913)	0.623
Chronic disease		
*None*	ref	
*One*	0.831 (0.631, 1.095)	0.188
*Multiple*	0.609 (0.474, 0.782)	<0.001
IADL		
*0 functional impairment)*	ref	
*1 functional impairment*	1.252 (0.963, 1.626)	0.093
*2 functional impairments*	1.968 (1.463, 2.647)	<0.001
*3 functional impairments*	2.505 (1.800, 3.486)	<0.001
*4 functional impairments*	2.713 (1.861, 3.956)	<0.001
*5 functional impairments*	5.916 (3.770, 9.283)	<0.001
Depression		
*No*	ref	
*Yes*	1.250 (1.028, 1.519)	0.025
Smoking		
*No*	ref	
*Yes*	0.937 (0.730, 1.203)	0.610
Drinking		
*No*	ref	
*Yes*	0.725 (0.578, 0.911)	0.006
Year		
*2011*	ref	
*2013*	0.933 (0.696, 1.250)	0.642
*2015*	1.104 (0.850, 1.434)	0.458
*2018*	1.886 (1.457, 2.441)	<0.001
*2020*	1.142 (0.808, 1.614)	0.453
Constant	0.320 (0.182, 0.562)	<0.001

CI – confidence interval, IADL – instrumental activities of daily living, OR – odds ratio, ref – reference

### Mediating effect of social activity between hearing aid use and cognitive function

**Figure 1:**
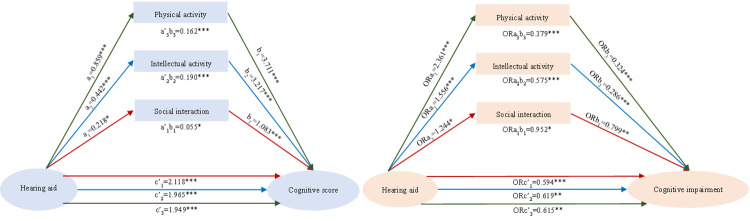
Mediating effect of social activities between hearing aid use and cognitive function. a/ORa quantifies hearing-aid use – social activity, a’ is standardised by a; c’/ORc’ give its direct effects on cognitive scores/impairment risk after social activity is controlled; b/ORb’ gives social activity’s effects on cognitive scores/impairment risk after hearing-aid use is controlled. *P < 0.1. **P < 0.05. ***P < 0.01.

After controlling for social interaction, intellectual participation, and physical activity, the direct effects of hearing aid use on cognitive scores were 2.118, 1.965, and 1.949, respectively, all statistically significant (**Figure 1**). Hearing aid use was significantly and positively associated with greater participation in social interaction (coefficient = 0.218), intellectual activities (coefficient = 0.442), and physical activity (coefficient = 0.859). In turn, participation in these activities was positively correlated with cognitive scores – for social interaction (coefficient = 1.083), intellectual activities (coefficient = 3.217), and physical activity (coefficient = 3.711). Consequently, the indirect effects of hearing aid use on cognitive scores, mediated by social interaction, intellectual engagement, and physical activity, were 0.055, 0.190, and 0.162, respectively. These mediating effects accounted for 2.53%, 8.75%, and 7.47% of the total effect.

After controlling for social interaction, participation in intellectual and physical activities, and hearing aid use, hearing aid use was associated with a 40.60%, 38.10%, and 38.50% reduction in the risk of cognitive impairment, respectively. Additionally, the use of hearing aids was significantly correlated with an increased likelihood of participation in social interactions (OR = 1.224), intellectual activities (OR = 1.556), and physical activities (OR = 2.361), with increases of 24.40%, 55.60%, and 136.10%, respectively. Furthermore, participation in social interactions (OR = 0.799), intellectual activities (OR = 0.286), and physical activities (OR = 0.324) was associated with a 20.10%, 71.40%, and 67.60% reduction in the risk of cognitive impairment, respectively. Thus, the use of hearing aids was also associated with a reduced risk of cognitive impairment through increased social interaction (OR = 0.952), participation in intellectual activities (OR = 0.575), and physical activity (OR = 0.379), with correlations of 4.80%, 42.50%, and 62.10%, respectively. These associations accounted for 8.62%, 53.57%, and 66.67% of the total observed effect.

### Heterogeneity analysis

#### Gender-based heterogeneity

**Figure 2:**
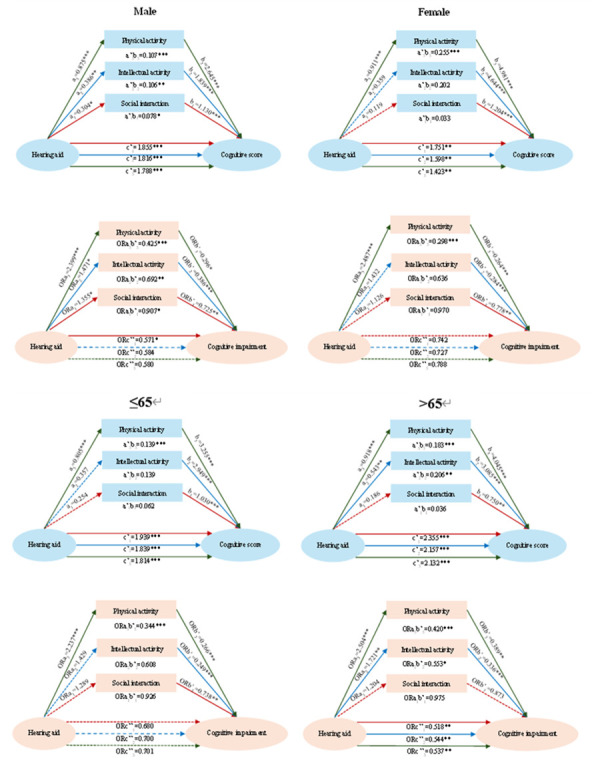
Heterogeneity analysis. a/ORa quantifies hearing-aid use – social activity, a’ is standardised by a; c’/ORc’ give its direct effects on cognitive scores/impairment risk after social activity is controlled; b/ORb’ give social activity’s effects on cognitive scores/impairment risk after hearing-aid use is controlled. *P < 0.1. **P < 0.05. ***P < 0.01.

Hearing aid use was associated with significant direct effects on cognitive scores for both men and women, after controlling for social interaction (men coefficient = 1.855; women coefficient = 1.751), intellectual activity (men coefficient = 1.816; women coefficient = 1.598), and physical activity (men coefficient = 1.788; women coefficient = 1.423), respectively (**Figure 2**). However, significant gender-based differences emerged in indirect effects. Among male participants, the indirect effects of hearing aid use on cognitive scores through social interaction (coefficient = 0.078), intellectual activity (coefficient = 0.106), and physical activity (coefficient = 0.107) were significant. In contrast, for women, only the pathway mediated by physical activity showed a significant indirect effect (coefficient = 0.255).

Among male participants, direct use of hearing aids was associated with a 42.90% lower likelihood of cognitive impairment (OR = 0.571). Regarding indirect effects, hearing aid use was associated with a 9.30%, 30.80%, and 57.50% decrease in the likelihood of cognitive impairment through participation in social interaction (OR = 0.907), intellectual activity (OR = 0.692), and physical activity (OR = 0.425). In contrast, among female participants, hearing aid use showed a significant indirect effect only through physical activity, with a 70.20% lower likelihood of cognitive impairment (OR = 0.298).

#### Age-based heterogeneity

After controlling for social interaction, intellectual activity, and physical activity, the direct effects of hearing aid use on cognitive scores were significantly greater for people aged >65 years (coefficient = 2.355 for social interaction; coefficient = 2.157 for intellectual activity; and coefficient = 2.132 for physical activity) compared with those aged ≤65 years (coefficient = 1.939 for social interaction; coefficient = 1.839 for intellectual activity; and coefficient = 1.814 for physical activity). In the younger cohort, hearing aid use had a significant indirect effect on cognitive scores only through physical activity (coefficient = 0.139). In contrast, people aged >65 years demonstrated significant indirect effects of hearing aid use on cognitive scores through both intellectual (coefficient = 0.206) and physical activity (coefficient = 0.183) (**Figure 2**).

After adjusting for social interaction, intellectual activity, and physical activity, the direct use of hearing aids was associated with a 48.20% (OR = 0.518), 45.60% (OR = 0.544), and 46.30% (OR = 0.537) lower likelihood of cognitive impairment among individuals aged ≥65 years, respectively. Regarding indirect effects, among younger individuals, the use of hearing aids was associated with a 65.60% lower likelihood of cognitive impairment, independent of physical activity (OR = 0.344). In contrast, for individuals aged >65 years, the use of hearing aids was associated with a 44.70% lower likelihood of cognitive impairment through participation in intellectual activities (OR = 0.553) and a 58.00% lower likelihood through physical activities (OR = 0.420).

## DISCUSSION

We examined the association between hearing aid use and cognitive function among middle-aged and older adults with hearing impairment in China, using representative national data from CHARLS. Additionally, we explored the mediating role of social activity. Our findings revealed that hearing aid use significantly improved cognitive function and reduced the risk of cognitive impairment in this population. Additionally, social activity was identified as a significant mediator, partially explaining how hearing aid use may help protect cognitive health among middle-aged and older adults. Furthermore, gender and age differences were observed in the mediating role of social activities. These results enhance our understanding of the relationship between hearing aid use and cognitive function.

Our analyses revealed that hearing-aid use was associated with a significant improvement in cognitive function and a 29.60% lower likelihood of cognitive impairment among hearing-impaired middle-aged and older adults, consistent with findings from previous studies [13,14]. Under the cognitive-load hypothesis, effortful listening usurps resources that would otherwise support executive control and memory encoding [14]; hearing aids relieve this burden by restoring audibility, thereby reallocating resources to higher-order tasks [34]. However, only 6.60% of the hearing-impaired older adult population in this study reported using hearing aids. Public campaigns should effectively highlight the cognitive benefits of hearing aid use to raise awareness among the general public and healthcare professionals. Clinicians should prioritise early fitting of hearing aids within comprehensive cognitive care protocols to ensure timely interventions. Policymakers must enhance access to hearing aids by expanding subsidies and insurance coverage for low-income and underserved older adults. These measures will reduce financial barriers and encourage adoption, ultimately improving hearing health and cognitive outcomes in this vulnerable population.

Beyond hearing aid use, age, sex, marital status, education, and IADL independently shaped cognition, as shown earlier [35–37]. Ageing-related cerebral atrophy and neurotransmitter depletion erode neural reserve [35]; oestrogen withdrawal post-menopause disproportionately compromises women’s trajectories [36]. Marriage mitigates loneliness-induced decline [37] and higher education proxies superior socioeconomic resources that enhance healthcare and nutrition access [38]. Integrated early hearing–cognitive screening, sex-specific interventions, strengthened social support, and equitable resource allocation are therefore recommended to attenuate mid- and late-life cognitive deterioration.

Hearing-aid use enhances cognition directly and, more importantly, indirectly, affecting middle-aged and older adults’ social lives [13,39–42]. Communication barriers commonly precipitate withdrawal and isolation, a well-established risk factor for cognitive decline [40,41]. Social-cognitive theory posits that auditory input shapes environmental perception and behavioural self-regulation [41]. Amplification, therefore, restores interactive capacity, fostering activities that stimulate and protect the brain. In our sample, the indirect pathway dominated: intellectual (mah-jongg, chess, cards) and physical (dance, fitness, qigong) activities mediated 42.50% and 62.10%, respectively, of the hearing aid’s effect on the risks of cognitive impairment, also explaining 7.47% and 8.75% of the total effect on cognitive scores. Intellectual games demand memory, attention and problem-solving [43]. Exercise boosts cerebral perfusion and plasticity [44]. Consequently, interventions should couple early hearing-aid fitting with organised programmes of intellectually and physically engaging pastimes to maximise cognitive gains and minimise the risk of impairment in hearing-impaired middle-aged and older adults.

The mediating effects of social activities on the relationship between hearing aid use and cognitive function vary by gender and age. Among men, all three activity domains – social interaction, intellectual pursuits, and physical exercise – significantly mediated the effect; in women, only physical activity was significant. Chinese middle-aged and older men tend to engage in structured, cognitively demanding, and interactive activities that provide both social and neural stimulation, whereas women are more likely to participate in emotionally supportive and informal interactions that offer relatively weaker cognitive activation [45]. Therefore, strategies should encourage men to engage in social activities while promoting diverse forms of physical activity for women to enhance cognitive function through hearing aid use.

Furthermore, our findings revealed significant age-related differences. Among people aged >65 years, hearing aids produced larger direct gains in cognitive scores and greater risk reduction than among those aged ≤65 years. Regarding indirect effects, younger people benefited primarily through physical activity, whereas the older group drew significant additional benefit from intellectual pursuits. This phenomenon may be attributed to the limitations in physical activity often faced by individuals aged >65 years, which predispose them to derive greater cognitive benefits from intellectual engagement compared to their younger counterparts with hearing impairment. Therefore, it is imperative that strategies be developed to encourage the use of hearing aids among older adults aged >65 years, while simultaneously promoting age-specific social activities. These activities should include a diverse range of intellectual pursuits for individuals aged ≥65 years and physical activities for younger seniors to optimise cognitive benefits.

### Limitations

This study has several important limitations that should be acknowledged. First, while data from five waves of CHARLS (2011, 2013, 2015, 2018, and 2020) were pooled for analysis, the cross-sectional design limits our ability to draw firm causal inferences regarding the relationship between hearing aid use and cognitive function. The nature of cross-sectional data means we cannot definitively determine whether hearing aid use prevents cognitive decline or if cognitively healthier individuals are more likely to utilise hearing aids.

Second, reliance on self-reported hearing impairment data may introduce bias, as individuals may misreport their hearing status for various reasons, including social desirability or lack of awareness. Future research should incorporate more objective assessment methods, such as audiometric testing, to enhance the reliability of the data and the validity of the conclusions.

Third, the study may be subject to residual confounding, as unmeasured variables likely influence both hearing impairment and cognitive function. Additionally, selection bias may arise from excluding participants with missing data, which could limit the generalizability of our findings. Participants who are lost to follow-up or who do not complete the survey may differ systematically from those who do, potentially skewing the results.

Furthermore, potential survivorship bias across CHARLS waves should be considered. Individuals with more severe cognitive impairment may not be represented in the later waves, leading to an underestimation of the true prevalence of cognitive decline among those with hearing impairment.

Lastly, cognitive function among middle-aged and older adults with hearing impairment is likely influenced by additional factors not captured in the CHARLS data, such as mental health status, socioeconomic factors, and lifestyle choices. This limitation indicates the need for a more comprehensive approach to understanding the complex interplay between hearing impairment and cognitive health.

## CONCLUSIONS

Hearing aid use significantly enhances cognitive function and decreases the risk of cognitive impairment among middle-aged and older adults with hearing impairment. Furthermore, hearing aid use indirectly enhanced cognitive function and reduced the risk of impairment by fostering social activities, with intellectual and physical activities showing the strongest mediating effects. Given the observed gender- and age-related variations, targeted strategies promoting hearing aid use and tailored social participation may further optimise cognitive function in this population.

## Additional material


Online Supplementary Document
