# Supplementary material for: Association between hearing aid use and cognitive function among Chinese middle-aged and older adults with hearing impairment: the mediating effect of social activity
Source: J Glob Health. 2026 May 22;16:04120. doi: 10.7189/jogh.16.04120 (PMC13196490; doi:10.7189/jogh.16.04120)
Supplement: Online Supplementary Document [file jogh-16-04120-s001.pdf]

Supplement to: Wang X, Gu X, Mao S, Zhang Z, Wang Z. Association between hearing aid use and cognitive function among Chinese middle-aged and older adults with hearing impairment: the mediating effect of social activity. J Glob Health. 2026;16:04120.

Supplementary table 1. Adherence to JoGH's GRABDROP guidelines items.

**JoGH guideline items**
1. Please list all papers published by each co-author in previous 3 years that were based on secondary analysis of a big data repository
Peng M, Shi X, Zhu L, **Wang Z**. Follow-up management service and health outcomes of hypertensive patients in China: A cross-sectional analysis from the national health service survey in Jiangsu province. Front Public Health. 2022 Jul 26;10:956711. doi: 10.3389/fpubh.2022.956711.<br>Zhu L, Peng M, Jiang L, **Wang Z**. Inequality of opportunity in health service utilization among middle-aged and elderly community-dwelling adults in China. Arch Public Health. 2023 Jan 25;81(1):13. doi: 10.1186/s13690-022-01010-1.<br>Wang Y, Jiang N, Shao H, **Wang Z**. Exploring unmet healthcare needs and associated inequalities among middle-aged and older adults in Eastern China during the progression toward universal health coverage. Health Econ Rev. 2024 Jun 27;14(1):46. doi: 10.1186/s13561-024-00521-7.<br>**Wang X**, Li S, **Zhang Z**, **Cui Z**, **Wang Z**. Association between widowhood and cognitive function among Chinese older adults with hearing impairment: the moderating effect of social support and participation. BMC Geriatr. 2024 Sep 17;24(1):764. doi: 10.1186/s12877-024-05358-7.
2. Please explain the key elements of your study design and the use of the available datasets that make your study an original scientific contribution
In our study, we employed a pooled cross-sectional data analysis approach, utilizing data from five waves of the China Health and Retirement Longitudinal Study (CHARLS). This methodology enabled us to conduct a comprehensive cross-sectional analysis that enhances our understanding of the relationship between hearing aid use and cognitive function among middle-aged and older adults.<br>Our study design incorporates several key elements. First, by integrating data from five different waves, we constructed a large-scale cross-sectional dataset. This mixed cross-sectional analysis allows us to capture a diverse snapshot of the population over time, enriching our findings and providing robust insights into the trends and patterns associated with hearing aid use and cognitive function.<br>Moreover, the CHARLS dataset encompasses a wide range of variables, including demographic information, health status, cognitive assessments, and details regarding hearing aid usage. This richness enables us to perform a detailed analysis while controlling for various confounding factors, thereby strengthening the validity of our results. Our investigation specifically focuses on the impact of hearing aid use on cognitive function—a critical area of research that has been underexplored. By concentrating on this relationship, we aim to contribute new knowledge to the fields of gerontology and cognitive health.

To ensure statistical rigor, we employed advanced statistical techniques suitable for pooled cross-sectional data, including regression analysis, to evaluate the associations between hearing aid use and cognitive performance. This methodological rigor enhances the reliability of our results and allows for more nuanced interpretations. Additionally, our study addresses significant gaps in the existing literature by exploring how hearing aid use may influence cognitive health in a representative population. The insights gained from our analysis can inform future research and public health interventions aimed at improving cognitive outcomes among older adults.

Understanding the relationship between hearing aids and cognitive function can have important implications for healthcare providers and policymakers. Our findings may support initiatives to promote hearing aid adoption, potentially enhancing cognitive health and overall quality of life for older individuals.

In summary, our mixed cross-sectional analysis utilizing data from five waves of CHARLS offers a novel contribution to the understanding of hearing aid use and cognitive function. By leveraging this extensive dataset, we aim to provide valuable insights that can inform both research and practical applications in the fields of aging and cognitive health.

3. Please list all publications that addressed similar research questions in the same dataset and indicate where you cited them in your paper

Hu Y, Peng W, Ren R, Wang Y, Wang G. Sarcopenia and mild cognitive impairment among elderly adults: The first longitudinal evidence from CHARLS. *J Cachexia Sarcopenia Muscle.* 2022;13(6):2944-2952.

Zhang X, Huang X, Du J, Zhang W, Gong L. Association between insufficient physical activity and the risk of cognitive impairment in middle-aged and elderly people in rural China. *Journal of Chongqing Medical University.*2024;49(07):919-924. (In Chinese)

MacKinnon, D. P., & Dwyer, J. H.. Estimating mediated effects in prevention studies. *Evaluation Review.*1993;17, 144-158.

MacKinnon, D. P.. Introduction to statistical mediation analysis. Mahwah, NJ: Erlbaum; 2008.

4. Please explain how you addressed multiple testing through an appropriately rigorous statistical threshold and indicate this in the methods section

To mitigate the risk of Type I errors, we employed a rigorous statistical threshold and made adjustments to our significance levels. Specifically, we used the Bonferroni correction method to adjust the p-values for multiple comparisons.

Methods Section

In the methods section of our manuscript, we detailed our approach to multiple testing as follows:

We conducted our analyses using STATA17.0. For each of the hypotheses tested, we calculated p-values based on linear regression, logistic regression.

To account for multiple testing, we applied the Bonferroni correction. The threshold for statistical significance was adjusted from the conventional alpha level of 0.1 to a more stringent level based on the number of tests conducted. Specifically, we calculated the adjusted p-value threshold by dividing 0.1 by the number of hypotheses tested.

By implementing this rigorous approach to multiple testing, we aimed to enhance the reliability of our findings and reduce the likelihood of false-positive results.

5. Please declare to what extent have AI chatbots been used in developing your paper and to which parts of the paper did they contribute
In the preparation of this manuscript, we utilized AI chatbots specifically for grammar and language refinement. These AI tools were employed in several key areas to enhance the quality of our work. First, they assisted in improving the overall clarity and readability of the text, ensuring that complex ideas were communicated more effectively. Additionally, the AI tools were instrumental in identifying and correcting grammatical errors, thereby enhancing sentence structure throughout the manuscript. They also contributed to maintaining consistency in terminology and phrasing across different sections of the paper, which helped create a more cohesive presentation of our findings.<br>It is important to emphasize that while the AI chatbots played a supportive role in the editing and refinement process, the core research ideas, analyses, and interpretations were developed by the authors themselves, reflecting our original contributions to the field. We acknowledge the assistance provided by these AI tools in enhancing the manuscript and appreciate the opportunity to clarify their role in the development of this paper.

Supplementary table 2. Multiple linear regression of cognitive scores among individuals with hearing impairment: excluding the effect of IADL and depression

	Coeff.	CI
Hearing aid use (ref: no)		
Yes	1.368***	[0.648, 2.089]
Age (years) (ref: 45–54)		
55–64	-1.720***	[-2.274, -1.165]
65-74	-3.013***	[-3.578, -2.448]
⩾75	-5.236***	[-5.886, -4.585]
Gender (ref: female)		
Male	3.004***	[2.488, 3.520]
Marital status (ref: others)		
Marry	1.801***	[1.302, 2.300]
Education (ref: non-illiterate)		
Illiterate	-2.389***	[-2.831, -1.947]
Household per capita consumption expenditure (Yuan) (ref: <5000)		
5000–9999	0.986***	[0.523, 1.448]
10000-19999	1.074***	[1.156, 2.090]
⩾20000	2.410***	[1.796, 3.024]
Self-rated health (ref: very good)		

Good	-0.069	[-1.016, 0.878]
Fair	0.008	[-0.785, 0.800]
Poor	-1.478***	[-2.310, -0.646]
Very poor	-2.221***	[-3.181, -1.262]
Chronic disease (ref: none)		
One chronic disease	0.387	[-0.175, 0.949]
Multiple chronic diseases	1.016***	[0.510, 1.522]
Smoke (ref: no)		
Yes	-0.330	[-0.820, 0.161]
Drink (ref: no)		
Yes	0.215	[-0.205, 0.634]
Year (ref: 2011)		
2013	0.478*	[-0.089, 1.046]
2015	-0.051	[-0.581, 0.479]
2018	-0.767***	[-1.309, -0.224]
2020	0.745**	[0.089, 1.402]
Cons	10.675***	[9.614, 11.735]

Note: *** implies p-value < 0.01, ** indicates p-value < 0.05, * implies p-value < 0.1

Supplementary table 3. Binary-logistic regression of cognitive impairment among individuals with hearing impairment: excluding the effect of IADL and depression

	OR	CI
Hearing aid use (ref: no)		
Yes	0.698*	[0.464, 1.050]
Age (years) (ref: 45–54)		
55–64	1.340**	[1.015, 1.769]
65-74	1.469***	[1.109, 1.946]
⩾75	1.018	[0.732, 1.416]
Gender (ref: female)		
Male	0.358***	[0.276, 0.463]
Marital status (ref: others)		
Marry	0.647***	[0.514, 0.815]
Education (ref: non-illiterate)		
Illiterate	1.578***	[1.265, 1.968]
Household per capita consumption expenditure (Yuan) (ref: <5000)		
5000–9999	0.732***	[0.588, 0.912]
10000-19999	0.759**	[0.596, 0.968]
⩾20000	0.536***	[0.389, 0.738]
Self-rated health (ref: very good)		
Good	1.550*	[0.939, 2.558]
Fair	1.262	[0.816, 1.951]
Poor	2.082***	[1.334, 3.250]
Very poor	2.241***	[1.371, 3.663
Chronic disease (ref: none)		
One chronic disease	0.866	[0.661, 1.134]
Multiple chronic diseases	0.667***	[0.523, 0.852]
Smoke (ref: no)		
Yes	0.905	[0.709, 1.156]
Drink (ref: no)		
Yes	0.706***	[0.565, 0.883]
Year (ref: 2011)		
2013	0.869	[0.652, 1.157]
2015	1.083	[0.838, 1.399]
2018	1.776***	[1.380, 2.284]
2020	0.969	[0.690, 1.360]
Cons	0.367***	[0.212, 0.635]

Note: *** implies p-value < 0.01, ** indicates p-value < 0.05, * implies p-value < 0.1

Supplementary table 4. Multiple linear regression of cognitive scores among individuals with hearing impairment: excluding aged 75 and above

	Coeff.(1)	CI(1)	Coeff.(2)	CI(2)
Hearing aid use (ref: no)				
Yes	1.386***	[0.568, 2.203]	1.247***	[0.450, 2.044]
Age (years) (ref: 45–54)				
55–64	-1.692***	[-2.254, -1.130]	-1.640***	[-2.188, -1.092]
65-74	-2.916***	[-3.495, -2.338]	-2.737***	[-3.304, -2.169]
Gender (ref: female)				
Male	2.954***	[2.353, 3.554]	2.583***	[1.993, 3.173]
Marital status (ref: others)				
Marry	1.877***	[1.262, 2.491]	1.634***	[1.031, 2.237]
Education (ref: non-illiterate)				
Illiterate	-2.287***	[-2.794, -1.780]	-2.045***	[-2.540, -1.549]
Household per capita consumption expenditure (Yuan) (ref: <5000)				
5000–9999	1.097***	[0.573, 1.620]	0.988***	[0.478, 1.498]
10000-19999	1.738***	[1.164, 2.311]	1.516***	[0.956, 2.076]
⩾20000	2.364***	[1.666, 3.063]	2.355***	[1.674, 3.036]
Self-rated health (ref: very good)				
Good	0.628	[-0.442, 1.699]	0.661	[-0.382, 1.803]
Fair	0.452	[-0.425, 1.329]	0.765*	[-0.091, 1.622]
Poor	-0.918*	[-1.844, 0.008]	0.245	[-0.677, 1.167]
Very poor	-1.724***	[-2.798, -0.651]	0.284	[-0.804, 1.373]
Chronic disease (ref: none)				
One chronic disease	0.574*	[-0.058, 1.206]	0.639**	[0.023, 1.256]
Multiple chronic diseases	0.975***	[0.406, 1.545]	1.215***	[0.659, 1.771]
IADL (ref: 0 functional impairment)				
1 Functional impairment	—	—	-0.807***	[-1.386, -0.227]
2 Functional impairment	—	—	-1.974***	[-2.717, -1.230]
3 Functional impairment	—	—	-2.805***	[-3.738, -1.871]
4 Functional impairment	—	—	-3.125***	[-4.183, -2.068]
5 Functional impairment	—	—	-5.307***	[-6.747, -3.868]
Depression (ref: no)				
Yes	—	—	-1.294***	[-1.726, -0.862]
Smoke (ref: no)				
Yes	-0.335	[-0.899, 0.229]	-0.324	[-0.874, 0.227]
Drink (ref: no)				

Yes	0.277	[-0.201, 0.754]	0.222	[-0.243, 0.687]
Year (ref: 2011)				
2013	0.412	[-0.220, 1.044]	0.221	[-0.395, 0.838]
2015	-0.063	[-0.652, 0.527]	-0.097	[-0.672, 0.477]
2018	-0.903***	[-1.509, -0.297]	-0.980***	[-1.571, -0.389]
2020	0.077	[-0.752, 0.905]	-0.160	[-0.968, 0.648]
Cons	10.081***	[8.881, 11.281]	10.982***	[9.791, 12.172]

Note: *** implies p-value < 0.01, ** indicates p-value < 0.05, * implies p-value < 0.1

Supplementary table 5. Binary-logistic regression of cognitive impairment among individuals with hearing impairment: excluding aged 75 and above

	OR(1)	CI(1)	OR(2)	CI(2)
Hearing aid use (ref: no)				
Yes	0.715	[0.456, 1.121]	0.717	[0.452, 1.136]
Age (years) (ref: 45–54)				
55–64	1.303*	[0.985, 1.722]	1.255	[0.946, 1.666]
65-74	1.382**	[1.039, 1.840]	1.260	[0.941, 1.687]
Gender (ref: female)				
Male	0.341***	[0.253, 0.458]	0.364***	[0.269, 0.492]
Marital status (ref: others)				
Marry	0.605***	[0.461, 0.792]	0.619***	[0.469, 0.817]
Education (ref: non-illiterate)				
Illiterate	1.743***	[1.363, 2.230]	1.640***	[1.276, 2.108]
Household per capita consumption expenditure (Yuan) (ref: <5000)				
5000–9999	0.714***	[0.559, 0.912]	0.732**	[0.570, 0.939]
10000-19999	0.677***	[0.515, 0.889]	0.718**	[0.544, 0.949]
⩾20000	0.598***	[0.423, 0.845]	0.585***	[0.410, 0.833]
Self-rated health (ref: very good)				
Good	1.362	[0.782, 2.370]	1.338	[0.766, 2.338]
Fair	1.201	[0.754, 1.914]	1.094	[0.683, 1.751]
Poor	1.907***	[1.181, 3.078]	1.375	[0.839, 2.252]
Very poor	2.017**	[1.184, 3.435]	1.087	[0.619, 1.907]
Chronic disease (ref: none)				
One chronic disease	0.912	[0.673, 1.235]	0.891	[0.654, 1.212]
Multiple chronic diseases	0.702**	[0.534, 0.924]	0.648***	[0.490, 0.857]
IADL (ref: 0 functional impairment)				
1 Functional impairment	—	—	1.279*	[0.961, 1.702]
2 Functional impairments	—	—	2.002***	[1.443, 2.778]
3 Functional impairments	—	—	2.553***	[1.737, 3.753]
4 Functional impairments	—	—	2.100***	[1.333, 3.306]
5 Functional impairments	—	—	6.320***	[3.562, 11.212]
Depression (ref: no)				
Yes	—	—	1.244**	[1.001, 1.547]
Smoke (ref: no)				
Yes	0.959	[0.728, 1.265]	0.978	[0.739, 1.295]
Drink (ref: no)				

Yes	0.711***	[0.554, 0.912]	0.727**	[0.564, 0.937]
Year (ref: 2011)				
2013	0.786	[0.569, 1.084]	0.828	[0.567, 1.150]
2015	1.062	[0.801, 1.408]	1.062	[0.797, 1.417]
2018	1.805***	[1.367, 2.382]	1.852***	[1.396, 2.460]
2020	1.142	[0.757, 1.723]	1.272	[0.839, 1.930]
Cons	0.413***	[0.227, 0.750]	0.355***	[0.192, 0.656]

Note: the reference group for the dependent variable comprises respondents without cognitive impairment.; *** implies p-value < 0.01, ** indicates p-value < 0.05, * implies p-value < 0.1

Supplementary table 6. Multiple linear regression of cognitive scores among individuals with hearing impairment: excluding aged 65 and above

	Coeff.(1)	CI(1)	Coeff.(2)	CI(2)
Hearing aid use (ref: no)				
Yes	1.587***	[0.504, 2.670]	1.498***	[0.445, 2.550]
Age (years) (ref: 45–54)				
55–64	-1.703***	[-2.270, -1.136]	-1.631***	[-2.183, -1.078]
Gender (ref: female)				
Male	2.283***	[1.454, 3.111]	1.972***	[1.161, 2.782]
Marital status (ref: others)				
Marry	1.463***	[0.565, 2.360]	1.229***	[0.353, 2.105]
Education (ref: non-illiterate)				
Illiterate	-1.794***	[-2.499, -1.088]	-1.525***	[-2.213, -0.836]
Household per capita consumption expenditure (Yuan) (ref: <5000)				
5000–9999	1.121***	[0.439, 1.802]	0.982***	[0.320, 1.645]
10000-19999	1.547***	[0.799, 2.295]	1.334***	[0.606, 2.061]
⩾20000	1.912***	[1.013, 2.812]	1.821***	[0.947, 2.695]
Self-rated health (ref: very good)				
Good	0.452	[-0.991, 1.895]	0.337	[-1.064, 1.738]
Fair	0.083	[-1.104, 1.271]	0.343	[-0.811, 1.498]
Poor	-1.342**	[-2.598, -0.086]	-0.133	[-1.375, 1.110]
Very poor	-2.355***	[-3.792, -0.919]	-0.288	[-1.738, 1.161]
Chronic disease (ref: none)				
One chronic disease	0.282	[-0.498, 1.063]	0.462	[-0.297, 1.222]
Multiple chronic diseases	0.781**	[0.072, 1.490]	1.099***	[0.408, 1.790]
IADL (ref: 0 functional impairment)				
1 Functional impairment	—	—	-0.926**	[-1.676, -0.176]
2 Functional impairment	—	—	-1.909***	[-2.911, -0.906]
3 Functional impairment	—	—	-3.395***	[-4.650, -2.141]
4 Functional impairment	—	—	-3.693***	[-5.299, -2.088]
5 Functional impairment	—	—	-5.565***	[-7.655, -3.475]
Depression (ref: no)				
Yes	—	—	-1.502***	[-2.056, -0.949]
Smoke (ref: no)				
Yes	-0.082	[-0.857, 0.692]	-0.150	[-0.093, 0.604]
Drink (ref: no)				
Yes	0.489	[-0.156, 1.094]	0.378	[-0.229, 0.986]

Year (ref: 2011)				
2013	1.015**	[0.205, 1.825]	0.821**	[0.033, 1.610]
2015	0.216	[-0.526, 0.959]	0.168	[-0.554, 0.891]
2018	-0.400	[-1.175, 0.374]	-0.475	[-1.227, 0.278]
2020	-0.373	[-1.804, 1.058]	-0.702	[-2.093, 0.690]
Cons	10.871***	[9.264, 12.478]	11.901***	[10.310, 13.491]

Note: *** implies p-value < 0.01, ** indicates p-value < 0.05, * implies p-value < 0.1

Supplementary table 7. Binary-logistic regression of cognitive impairment among individuals with hearing impairment: excluding aged 65 and above

	OR(1)	CI(1)	OR(2)	CI(2)
Hearing aid use (ref: no)				
Yes	0.683	[0.370, 1.261]	0.666	[0.356, 1.246]
Age (years) (ref: 45–54)				
55–64	1.230	[0.925, 1.635]	1.186	[0.888, 1.584]
Gender (ref: female)				
Male	0.345***	[0.224, 0.532]	0.363***	[0.233, 0.565]
Marital status (ref: others)				
Marry	0.583***	[0.389, 0.872]	0.590**	[0.390, 0.892]
Education (ref: non-illiterate)				
Illiterate	1.901***	[1.343, 2.693]	1.800***	[1.262, 2.567]
Household per capita consumption expenditure (Yuan) (ref: <5000)				
5000–9999	0.715**	[0.516, 0.991]	0.743*	[0.533, 1.036]
10000-19999	0.674**	[0.467, 0.973]	0.708*	[0.486, 1.030]
⩾20000	0.620**	[0.393, 0.980]	0.636*	[0.399, 1.012]
Self-rated health (ref: very good)				
Good	1.428	[0.629, 3.242]	1.467	[0.642, 3.354]
Fair	1.401	[0.692, 2.839]	1.306	[0.640, 2.667]
Poor	1.969*	[0.952, 4.071]	1.389	[0.658, 2.928]
Very poor	2.535**	[1.161, 5.536]	1.405	[0.621, 3.177]
Chronic disease (ref: none)				
One chronic disease	0.902	[0.611, 1.333]	0.844	[0.567, 1.256]
Multiple chronic diseases	0.834	[0.586, 1.186]	0.748	[0.522, 1.072]
IADL (ref: 0 functional impairment)				
1 Functional impairment	—	—	1.186	[0.809, 1.737]
2 Functional impairments	—	—	2.146***	[1.383, 3.332]
3 Functional impairments	—	—	3.000***	[1.795, 5.015]
4 Functional impairments	—	—	2.255**	[1.134, 4.485]
5 Functional impairments	—	—	5.544***	[2.414, 12.729]
Depression (ref: no)				
Yes	—	—	1.333**	[0.997, 1.781]
Smoke (ref: no)				
Yes	1.010	[0.679, 1.503]	1.058	[0.767, 1.585]
Drink (ref: no)				
Yes	0.721*	[0.517, 1.006]	0.737*	[0.524, 1.035]

Year (ref: 2011)				
2013	0.546***	[0.353, 0.844]	0.579**	[0.372, 0.902]
2015	0.815	[0.566, 1.171]	0.806	[0.556, 1.169]
2018	1.396*	[0.974, 1.999]	1.458**	[1.010, 2.104]
2020	1.165	[0.558, 2.429]	1.334	[0.637, 2.794]
Cons	0.415**	[0.176, 0.979]	0.343**	[0.142, 0.830]

Note: the reference group for the dependent variable comprises respondents without cognitive impairment.; *** implies p-value < 0.01, ** indicates p-value < 0.05, * implies p-value < 0.1

Supplementary table 8. Bootstrap: the mediating role of social activities between hearing aid use and cognitive scores

	Social interaction		Intellectual activity		Physical activity	
	Coeff.	*CI*	*Coeff.*	*CI*	*Coeff.*	*CI*
Indirect effect	0.055*	[-0.010, 0.131]	0.190***	[0.055, 0.326]	0.162***	[0.075,0.250]
Direct effect	2.118***	[1.271,2.945]	1.958***	[1.111, 2.805]	1.949***	[1.111, 2.787]
Total effect	2.174***	[1.340,3.000]	2.174***	[1.346, 3.002]	2.174***	[1.346, 3.002]
a’	0.051*	[-0.009,0.111]	0.058***	[0.017, 0.099]	0.044***	[0.021, 0.066]
b	1.083***	[0.684,1.500]	3.284***	[2.768, 3.800]	3.711***	[2.820, 4.602]

Note: *** implies p-value < 0.01, ** indicates p-value < 0.05, * implies p-value < 0.1

Supplementary table 9. Bootstrap: gender heterogeneity of mediating effects of social activities between hearing aid use and cognitive scores

	Male						Female					
	Social interaction		Intellectual activity		Physical activity		Social interaction		Intellectual activity		Physical activity	
	Coeff.	*CI*	*Coeff.*	*CI*	*Coeff.*	*CI*	*Coeff.*	*CI*	*Coeff.*	*CI*	*Coeff.*	*90%CI*
Indirect effect	0.078*	[-0.016, 0.173]	0.106*	[-0.012, 0.225]	0.107**	[0.021, 0.193]	0.034	[-0.092, 0.159]	0.202	[-0.080, 0.484]	0.255**	[0.060, 0.449]
Direct effect	1.855***	[0.888, 2.822]	1.820***	[0.839, 2.801]	1.788***	[0.815, 2.762]	1.751**	[0.364, 3.139]	1.552**	[0.130, 2.973]	1.423**	[0.227, 2.819]
Total effect	1.936***	[0.971, 2.900]	1.936***	[0.971, 2.900]	1.936***	[0.971, 2.900]	1.785**	[0.408, 3.163]	1.785**	[0.408, 3.163]	1.785**	[0.408, 3.163]
a’	0.069*	[-0.005, 0.143]	0.055*	[-0.004, 0.115]	0.040***	[0.013, 0.068]	0.028	[-0.073, 0.129]	0.043	[-0.017, 0.103]	0.051***	[0.013, 0.090]
b	1.130***	[0.585, 1.676]	1.923***	[1.299, 2.547]	2.645***	[1.367, 3.923]	1.204***	[0.592, 1.815]	4.679***	[3.800, 5.558]	4.981***	[3.722, 6.240]

Note: *** implies p-value < 0.01, ** indicates p-value < 0.05, * implies p-value < 0.1

Supplementary table 10. Bootstrap: age heterogeneity of mediating effects of social activities between hearing aid use and cognitive scores

	≤65						>65					
	Social interaction		Intellectual activity		Physical activity		Social interaction		Intellectual activity		Physical activity	
	Coeff.	*CI*	*Coeff.*	*CI*	*Coeff.*	*CI*	*Coeff.*	*CI*	*Coeff.*	*CI*	*Coeff.*	*CI*
Indirect effect	0.062	[-0.049, 0.172]	0.139	[-0.078, 0.355]	0.139**	[0.015, 0.263]	0.036	[-0.031, 0.102]	0.206**	[0.048, 0.364]	0.183***	[0.059, 0.307]
Direct effect	2.084***	[0.985, 3.183]	1.996***	[0.864, 3.128]	1.957***	[0.853, 3.062]	2.321***	[1.173, 3.469]	2.112***	[0.946, 3.278]	2.101***	[0.939, 3.263]
Total effect	2.147***	[1.048, 3.246]	2.147***	[1.048, 3.246]	2.147***	[1.048, 3.246]	2.357***	[1.217, 3.498]	2.357***	[1.217, 3.498]	2.357***	[1.217, 3.498]
a’	0.054	[-0.038, 0.147]	0.045	[-0.024, 0.114]	0.041**	[0.006, 0.076]	0.050	[-0.029, 0.130]	0.069***	[0.018, 0.119]	0.046***	[0.016, 0.075]
b	1.131***	[0.579, 1.683]	3.100***	[2.455, 3.745]	3.356	[2.249, 4.463]	0.705**	[0.126, 1.285]	3.002***	[2.226, 3.778]	4.010***	[2.677, 5.343]

Note: *** implies p-value < 0.01, ** indicates p-value < 0.05, * implies p-value < 0.1
